# The Influence of Cognitive Load on Empathy and Intention in Response to Infant Crying

**DOI:** 10.1038/srep28247

**Published:** 2016-06-16

**Authors:** Daiki Hiraoka, Michio Nomura

**Affiliations:** 1 Graduate School of Education, Kyoto University, Yoshida Hon-machi, Sakyo-ku, 606-8501, Kyoto, Japan

## Abstract

Many studies have explored risk factors for child maltreatment, but little research has focused on situational risk factors such as cognitive load, which involves within-individual fluctuation. The current study sought to determine whether cognitive load led to within-individual changes in intention in response to infant crying. The study also sought to ascertain whether state empathy, empathic concern (EC), and personal distress mediated or moderated this relationship. Sixty-six participants completed a memory task (remembering meaningless, two- or eight-letter, English alphabet string), during which they were required to keep these letters in mind while hearing infant crying (or a tone). Subsequently, participants rated questions concerning state empathy and intention in response to the crying (i.e., intentions involving caregiving, neglect, or physical abuse). Results showed that cognitive load reduced caregiving intention and increased intention to perpetrate neglect. In addition, EC mediated the relationship between cognitive load and intention to provide care or perpetrate neglect. Moreover, cognitive load interacted with state empathy to predict intention to provide care or perpetrate neglect. These findings highlighted the importance of focusing on situational cognitive risk factors for child maltreatment and elucidated the role of state empathy as a mediator or moderator in child maltreatment research.

Reports of child maltreatment have become more frequent over the last 12 years^1^. Infant and child maltreatment has severe, deleterious short- and long-term effects on children’s cognitive, socioemotional, and behavioral development and physical wellness^2^, and in the worst cases, can lead to death^3^. Therefore, it is critical to explore the causes and mechanisms underlying child maltreatment.

Infants often cry to communicate discomfort or the need for help from caregivers, who are usually motivated to eliminate the infant’s discomfort; however, many studies have suggested that infant crying is one of the factors that lead to abusive behavior, physical abuse, and neglect^4^. Previous studies have suggested that infant crying causes affective and physiological responses^5^,^6^, which are known to be influenced by individual variations in psychobiological factors such as personality traits^7^, depression^8^, and oxytocin receptor gene polymorphism^9^. However, these factors are dispositional, and few studies have examined between-situation and within-individual differences in responses to infant crying.

Previous studies examining risk factors for child maltreatment have focused mainly on between-individual dispositional and demographic factors (i.e., characteristics of abusive parents, delayed development in children, and socioeconomic status in families^10^); therefore, this study focused on the effects of cognitive load on self-regulation as a situational factor involving momentary within-individual fluctuation. Self-regulation involves the ability to control or override one’s thoughts, emotions, urges, and behavior^11^. Self-regulation can be temporarily impaired by situational factors such as cognitive load or ego depletion^11^ and a lack of self-regulation leads to less helping behavior^12^,^13^ or intimate partner violence^14^,^15^. Therefore, we hypothesized that cognitive load would reduce the intention to help and increase the intention to perpetrate maladaptive behavior in childrearing situations.

Empathy, which is thought to motivate prosocial behavior or inhibit aggression^16^, has been assumed to be influenced by cognitive factors^17^,^18^. Empathy involves the ability to understand or make inferences based on others’ experience and consists of cognitive factors, which allow people to take another person’s perspective, and affective factors, which enable people to experience another person’s emotions vicariously^19^. Furthermore, affective factors have been classified into empathic concern (EC) and personal distress (PD). Both EC and PD are aroused by the perception of discomfort in others^19^: EC involves sensitivity to or sympathy for another person’s discomfort, whereas PD involves experiencing discomfort or conflict. Furthermore, in state empathy, both EC and PD, aroused by infant crying, have been shown to positively predict intention to provide care^20^. Interestingly, cognitive load impairs subjective empathy (EC) and leads to dissociation of the activation of brain areas involved in empathy such as the amygdala and medial prefrontal cortex^21^, while cognitive factors do not affect PD^17^. In light of these observations, we predicted that cognitive load would influence empathy, impairing EC in particular, and this change in state empathy would predict maladaptive intention in response to infant crying.

## Current Research

This study sought to 1) determine whether cognitive load would lead to within-individual changes in intention in response to infant crying and 2) examine the mediation (or moderation) effects of two types of empathy. We hypothesized that cognitive load would negatively influence intention in response to infant crying (Hypothesis 1). We also hypothesized that cognitive load-related changes in intention in response to infant crying would be mediated by state empathy (Hypothesis 2).

## Results

### Preliminary Analyses: Memory Task Scores

Each memory task score was calculated by dividing the number of correct responses by the number of letter strings presented (low load condition: 2; high load condition: 8). In the low cognitive load condition, the mean proportion of correct responses was 0.99 (*SD* = 0.04) for infant crying and 0.98 (*SD* = 0.05) for the tone. In the high load condition, the mean proportions of correct responses were 0.48 (*SD* = 0.13) for infant crying and 0.44 (*SD* = 0.13) for the tone. A two-way repeated measures analysis of variance (ANOVA) with cognitive load and sound condition as factors revealed significant main effects of cognitive load (*F* (1,0 65) = 1,335.93, *p* < 0.001, 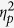 = 0.95), sound (*F* (1,0 65) = 5.83, *p* = 0.019, 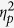 = 0.08) and an interaction between the two, (*F* (1, 65) = 4.28, *p* = 0.043, 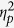 = 0.06). Post-hoc tests, using Bonferroni correction, indicated a significantly greater decrease in scores with the tone relative to that of infant crying (*t* (65) = 3.02, *p* = 0.004, *d* = 0.28) in the high cognitive load condition. These results showed that the memory task was significantly more difficult in the high cognitive load condition relative to that of the low cognitive load condition. Additionally, the results showed that the difficulty of the memory task was significantly greater with the tone compared to infant crying. This was an unexpected result as previous research^22^ reported that participants had the least correct trials on the working memory task when hearing infant crying compared to other noises. Although we cannot convincingly explain this result, the purpose of using the tone was simply to prevent participants from habituating themselves to repetitive infant crying and it was unlikely that the result influenced the following main results.

### Differences in Responses to Infant Crying between Cognitive Load Conditions

Table 1 presents descriptive statistics for state empathy and intention in response to infant crying for each condition. A paired *t* test, with Bonferroni correction, showed significant differences in mean ratings for EC (*t* (65) = 3.58, *p* = 0.003, *d* = 0.42) and intention to provide care (*t* (65) = 4.45, *p* < 0.001, *d* = 0.34) and perpetrate neglect (*t* (65) = 4.14, *p* < 0.001, *d* = 0.34) between cognitive load conditions. In the high load condition, levels of EC and intention to provide care were significantly lower and the level of intention to perpetrate neglect was significantly higher relative to those in the low load condition. No significant differences were observed with respect to PD (*t* (65) = 1.56, *p* = 0.123, *d* = −0.16) or intention to perpetrate physical abuse (*t* (65) = 1.83, *p* = 0.358, *d* = 0.14). These results showed that high cognitive load decreased EC and intention to provide care and increased intention to perpetrate neglect.

### Relationship between Intention and State Empathy

We created a multilevel hierarchical regression model to examine the effects of cognitive load, state empathy, EC, and PD on intention in response to infant crying. The intraclass correlation coefficients for intention to perpetrate neglect and physical abuse and provide care were 0.69, 0.66, and 0.72, respectively, which were larger effect sizes and provided evidence for a hierarchical structure^23^,^24^.

### Effects of Cognitive Load and State Empathy on Intention to Provide Care

**Figure 1:**
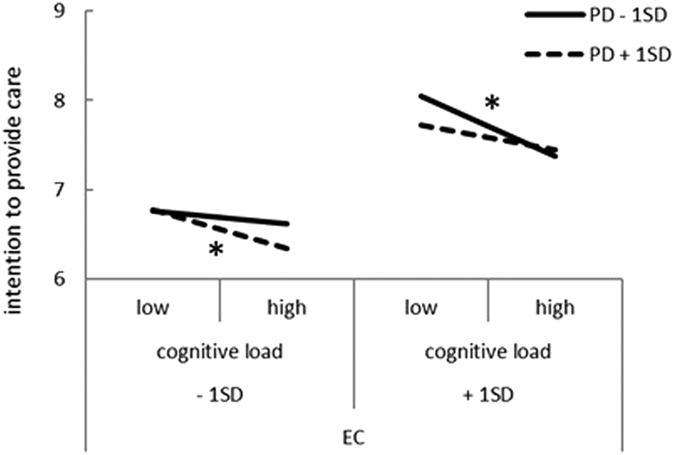
Interactive effect of cognitive load, EC, and PD on intention to provide care. The graph illustrates the result of simple slopes for the association between cognitive load and intention to provide care according to EC and PD. EC: empathic concern; PD: personal distress.

First, the effects of cognitive load and state empathy on intention to provide care were examined using multilevel analysis (Table 2A). Model 1 included only social desirability. Cognitive load, EC, and PD were then added as predictors in Model 2 (intercept-only model), which resulted in improved fit (*χ*^2^ [3] = 35.43, *p* < 0.001); the inclusion of random slopes for EC and PD in Model 3 also improved fit (*χ*^2^ [3] = 18.89, *p* = 0.002). Interactions between cognitive load, EC, and PD were added in Model 4 (intercept-only model) and improved fit further (*χ*^*2*^ [1] = 15.97, *p* < 0.001). Model 5, which included random slopes for EC and PD, showed further improved fit (*χ*^2^ [5] = 24.02, *p* < 0.001). Ultimately, Model 5 was adopted. In Model 5, EC predicted, and high cognitive load decreased, intention to provide care. In addition, the interaction between cognitive load, EC, and PD was significant, and we performed simple slope analysis to assess the significance of cognitive load slopes for EC and PD one standard deviation above and below the sample mean. The results revealed that cognitive load predicted a reduction in intention to provide care only when EC was high and PD was low (*β* = −0.61, *p* = 0.034), or when EC was low and PD was high (*β* = −0.67, *p* = 0.011; Fig. 1). This shows that the rating of intention of providing care estimated by low EC and high PD, and also high EC and low PD, decreased due to cognitive load.

### Effects of Cognitive Load and State Empathy on Intention to Perpetrate Neglect

**Figure 2:**
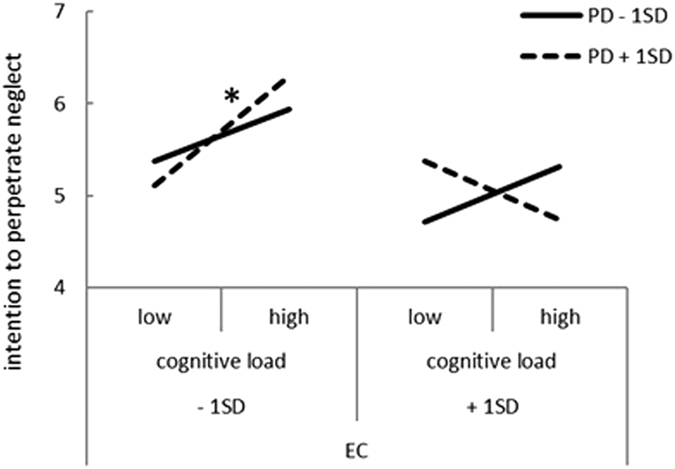
Interactive effect of cognitive load, EC, and PD on intention to perpetrate neglect. The graphs illustrate the results of simple slopes for the association between cognitive load and intention to perpetrate neglect according to EC and PD. EC: empathic concern; PD: personal distress.

In a similar analysis, we assessed the effects of cognitive load and state empathy on intention to perpetrate neglect (Table 2B). Social desirability was added in Model 1, and cognitive load, EC, and PD were added in Model 2 (intercept-only model). The fit of Model 2 was good relative to that of the previous model (*χ*^*2*^ [3] = 24.25, *p* < 0.001). Random slopes for EC and PD were included in Model 3, which improved fit (*χ*^*2*^ [5] = 30.61, *p* < 0.001). Adding the interactions between cognitive load and EC and cognitive load and PD in Model 4 (intercept-only model) resulted in further improvement in fit (*χ*^*2*^ [1] = 10.27, *p* = 0.001). Random slopes for EC and PD were then added in Model 5, which improved fit still further (*χ*^*2*^ [5] = 22.79, *p* < 0.001). In Model 5, cognitive load significantly increased and EC negatively predicted intention to perpetrate neglect. There was a significant interaction between cognitive load, EC, and PD, which affected intention to perpetrate neglect. Simple slope analysis revealed that associations between cognitive load and intention to perpetrate neglect were evident only when EC was low and PD was high (*β* = 1.20, *p* < 0.001; Fig. 2). This showed that the rating of intention of perpetrating neglect estimated by low EC and high PD increased due to cognitive load.

### Effects of Cognitive Load and State Empathy on Intention to Perpetrate Physical Abuse

The effects of cognitive load and state empathy on intention to perpetrate physical abuse were examined (Table 2C). The distribution of intention to perpetrate physical abuse was highly skewed (*skewness* = 1.49), and we used negative binomial regression in a multilevel model. Model 1 included only social desirability. Model 2 (intercept-only model), included cognitive load, EC, and PD, and the inclusion of random slopes for EC and PD (Model 3), interactions between cognitive load and EC and cognitive load and PD (Model 4: intercept-only model), and Model 5 including random slopes for EC and PD did not improve model fitness (*p*s > 0.53). The effects of cognitive load, EC, and PD on intention to perpetrate physical abuse were not confirmed.

### Mediation Analyses

Mediation analysis^25^ was conducted to assess the mediation effect of EC. PD and intention to perpetrate physical abuse were not included in the mediation analysis, because paired *t* tests comparing cognitive load conditions revealed no significant differences, and direct and mediation effects did not require consideration.

**Figure 3:**
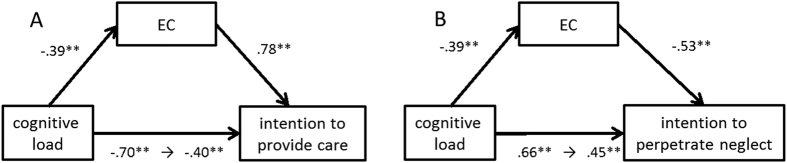
Mediation models. The left model shows that EC mediated the relationship between cognitive load and intention to provide care (Fig. 3A). The right demonstrates a similar effect on intention to perpetrate neglect (Fig. 3B). EC: empathic concern; PD: personal distress.

The mediation model predicting intention to provide care is presented in Fig. 3A. In this model, we provided cognitive load as the independent variable, EC as mediator, and intention to provide care as the dependent variable. Cognitive load and EC significantly predicted the intention to provide care as shown by the earlier multilevel regression analyses. Cognitive load also predicted EC (*β* = −0.39, *p* < 0.001). When cognitive load and EC were entered in a model predicting intention to provide care, the effect of cognitive load reduced (but it was still significant, *β* = −0.40, *p* < 0.001), and EC remained significant (*β* = 0.78, *p* < 0.001). A bootstrapping procedure^26^ tested EC as mediator with n = 2,000 resamples. This technique yielded a 95% bootstrap confidence interval (CI) that did not include zero [−0.51 to −0.13], suggesting that EC significantly mediated the effect of cognitive load on intention to provide care.

In a similar analysis, cognitive load predicted intention to perpetrate neglect. Figure 3B shows the mediation model predicting intention to perpetrate neglect. In this model, we provided cognitive load as the independent variable, EC as mediator, and intention to perpetrate neglect as the dependent variable. When cognitive load and EC were entered in a model predicting intention to perpetrate neglect, the effect of cognitive load reduced (but it was still significant; *β* = 0.45, *p* < 0.001), and EC remained significant (*β* = −0.53, *p* < 0.001). A bootstrapping procedure tested EC as mediator with n = 2,000 resamples. This technique yielded a 95% bootstrap confidence interval (CI) that did not include zero [0.07 to 0.43], suggesting that EC significantly mediated the effect of cognitive load on intention to perpetrate neglect.

## Discussion

This study sought to determine whether cognitive load would lead to within-individual changes in intention in response to infant crying and examine the mediation (or moderation) effects of two types of empathy. Hypotheses were generally supported in that 1) cognitive load decreased intention to provide care, and 2) EC mediated the relationship between cognitive load and intention in response to infant crying. In addition, the interaction between EC, PD, and cognitive load predicted intention to provide care and perpetrate neglect. To the best of our knowledge, this was the first study to demonstrate that situational cognitive factors increase the risk of within-individual maladaptive child caring practices, and empathy plays a significant role in these processes. Details and directions for future research are discussed below.

Cognitive load decreased intention to provide care and increased intention to perpetrate neglect, which is consistent with previous findings indicating that deficits in cognitive resources led to a reduction in helping behavior^12^,^13^. Notably, high cognitive load also decreased EC, and this change mediated the effects of cognitive load in decreasing intention to provide care and increasing intention to perpetrate neglect. These results are consistent with those of recent studies indicating that individuals experience difficulty in empathizing with others under cognitive load^21^, and state empathy predicts intention to provide care in response to infant crying^20^. These changes in EC and intention, resulting from cognitive load, can be explained in terms of incompetence in self-regulation. Self-regulation can be defined as the ability to override unwanted or maladaptive impulses, emotions, and behaviors^11^ and is known to be impaired by temporary situational factors such as cognitive load^11^. In this study, incompetence in self-regulation could be considered to have impaired EC, leading to a reduction in motivation to care for the crying infant.

This study showed that cognitive load affected EC but not PD; this is in line with previous findings^17^ suggesting that PD precedes EC and only EC is regulated by cognitive ability. Interestingly, PD interacted with EC and cognitive load in intention to provide care or perpetrate neglect. Graziano and Habashi^17^ proposed a four-dimensional (EC [high/low] × PD: [high/low]) system in which individuals with low EC and high PD were characterized as highly self-focused. From this perspective, it is reasonable to assume that cognitive load negatively predicted intention to provide care and positively predicted intention to perpetrate neglect when situational EC and PD were low and high, respectively. Previous studies^17^,^27^ suggested that low EC and high PD motivate egoistic behavior and it seems reasonable that the rating of intention of providing care, estimated by low EC and high PD decreased and intention to perpetrate neglect estimated by low EC and high PD increased due to cognitive load in this study. On the other hand, the state of high EC and low PD motivates altruistic behavior and this seems contradictory in that cognitive load affected the state of low EC and high PD in the same manner. However, Fig. 1 showed that in the low load condition, high EC and low PD predicted the highest rating of intention to provide care. Therefore, the effects of cognitive load seem to actualize the risk of low EC and high PD and diminish the merit of high EC and low PD. In other words, it is possible that people who normally empathize children possess an element of danger in high-risk situations, and the current results suggest a need to consider the importance of situational factors in future studies and clinical settings.

It should be noted that intention to provide care in response to infant crying has been examined as a mechanism in studies that aimed to determine how infant crying leads to caregiving in usual parent-infant relationships; we expanded this to include intention to perpetrate neglect and physical abuse, because there is also a risk that infant crying could lead to abusive behavior^4^. The result indicating that EC positively predicted intention to provide care supports the findings of previous studies^20^, and it is likely that EC was negatively correlated with intention to perpetrate neglect. Lin and McFatter^20^ reported that PD positively predicted intention to provide care, while the results of the current study showed that it did not predict this intention; rather, it was negatively associated with intention to provide care and positively associated with intention to perpetrate neglect in interactions involving cognitive load and EC. This discrepancy could be due to the possibility that caregiving derived from high PD would be merely one strategy to solace one’s own discomfort, not the infant’s, as the egoistic motivation and to reduce one’s own discomfort, although sometimes promote helping behavior^28^. This is speculative and needs to be confirmed empirically, and therefore, the examination of intention in response to infant crying, using various ecologically valid options, would be of interest in future research. Further studies should examine objectivity in the intention to perpetrate physical abuse, using behavioral and physiological indices. The results of this preliminary study revealed that cognitive load impaired motivation to care for crying infants, and state empathy played an important role in this relationship. Future research should also expand on the current findings by examining other situational factors, such as ego depletion, stressors, or alcohol intoxication, which impair self-regulation^11^, or interactive or cumulative relationships between situational factors and other dispositional or demographic factors identified in previous studies.

The study was subject to some limitations. The sample consisted of female university students who were not parents. Some studies have shown that mothers respond to infant crying in a unique manner^29^,^30^, however, other research showed that ratings of infant distress, EC, and intention to provide care did not differ between parents and nonparents^20^, and examining students holds some value, in that they are potentially future parents^7^ and the child-minder who is not yet a parent is at greatest risk of perpetrating severe abuse^31^. Nevertheless, the assessment of real mother-child relationships, using this paradigm, would be valid in future studies. It should be noted that this study also has clinical implications for child maltreatment intervention and prevention efforts. The results indicating that cognitive situational factors could temporarily increase maladaptive emotion or behavior directed toward infants is useful in understanding child abuse perpetrators. For example, Yusainy and Lawrence^32^ suggested that brief mindfulness meditation reduced aggression following the depletion of cognitive resources, and the results of the current study are expected to link such fundamental research to clinical implications in future.

In conclusion, this study sought to determine whether cognitive load would lead to within-individual changes in intention in response to infant crying and ascertain whether EC and PD mediated or moderated this relationship. The results revealed that cognitive load reduced EC and intention to provide care and increased intention to perpetrate neglect; more importantly, EC mediated the relationships between cognitive load and intention to provide care and perpetrate neglect. Moreover, cognitive load interacted with state empathy to predict intention to provide care and perpetrate neglect. These findings revealed the importance of focusing on situational cognitive risk factors for child maltreatment and the role of state empathy as a mediating or moderating variable in child maltreatment research.

## Methods

### Participants

Sixty-six female college students (mean age: 21.80 ± 1.63 years) with no experience in raising children participated in the study. Only women were recruited, because responses to infant crying are likely to differ between men and women^7^, and 60% of maltreatment is perpetrated by birth mothers^4^. Furthermore, violence perpetrated by mothers, rather than fathers, could have deleterious effects on children’s development^33^. Targeting only women was considered valid for these reasons. The study was approved by the review boards of Kyoto University and carried out in accordance with the approved guidelines. All participants provided written informed consent.

### Procedure

Participants completed a memory task while listening to sound stimuli, infant cries, or tones. Stimuli were presented under two within-participants conditions: low (remembering a meaningless, two-letter English alphabet) and high (remembering a meaningless, eight-letter English alphabet string) cognitive load. At the end of the blocks in each condition, participants rated their state empathy and intention in response to the infant crying stimulus. Subsequent to the memory task, participants completed a questionnaire.

### Memory Task: Infant Crying Paradigm

#### Sound stimuli

Recordings of spontaneously crying infant were created at home by the mother of one child aged 7 months. Four sound files of 30 s in duration were extracted from the recordings. The mean maximum fundamental frequency was 498.8 Hz, and the mean volume was 72.03 dB. The tones, which involved the same fundamental frequency and volume as the infant crying stimulus, were used to prevent participants from becoming habituated to repetitive infant crying stimulation. Tones were created and edited using Audacity 2.0.6^34^, and acoustic analysis was performed using Praat 5.4.04^35^. Sound stimuli were presented using loudspeakers (a525 2.1, Dell).

#### Task procedure

A meaningless English alphabet string to be memorized was presented on the display for 3 s, followed by a retention period lasting 30 s, during which the sound of an infant crying (or a tone) was presented. Subsequent to the retention period, participants recalled the memorized meaningless English alphabet string and provided a response using a keyboard. The recall score was calculated by counting the number of letters that were recalled in the correct serial order from the head of the string until the appearance of the first wrong letter and calculating the proportion of correct letters. Participants then received feedback regarding the score.

The above procedure constituted one trial, and one block consisted of eight trials. In each block, infant crying and tone stimuli were each presented four times in random order. Infant crying was different along a block and the same across different blocks. The tone was used repetitively along and across each block. Cognitive load was manipulated within subjects and between blocks. There were four blocks (two low-load and two high-load condition blocks) separated by 2 min of rest. The order of high- and low-load blocks was alternated.

#### State empathy and intention questionnaire

Intention in response to infant crying was rated at the end of each block. Each item was listed in Table 3. Intention in response to infant crying consisted of state EC, state PD, intention to provide care, intention to perpetrate physical abuse, and intention to perpetrate neglect. A previous study^20^ rated intention to provide care but did not rate intention to perpetrate physical abuse or neglect in response to infant crying. Although motivation resulting from PD predicts intention to provide care if there is only one response option^20^, it was possible that PD motivated other actions^28^. Therefore, physical abuse and neglect options were provided in the current study. Based on Lin and McFatter^20^, questionnaire items concerning intention to provide care consisted of two items (“I felt like picking up the baby” and “I felt like changing the diaper or bottle feeding the baby”). EC and PD were each represented by one item (EC: “I felt concerned for the baby”; PD: “The longer I listened to the crying, the more helpless and frustrated I felt”). Intention to perpetrate physical abuse and neglect were each represented by two items (physical abuse: “I want to slap the baby” and “I want to shake the baby strongly”; neglect: “I want to leave the baby alone” and “I want to go to another room”). All responses were provided using five-point Likert scales ranging from 1 (does not describe me well) to 5 (describes me very well). The *ω* coefficients for items concerning intention to provide care, perpetrate physical abuse, and perpetrate neglect were 0.81, 0.76, and 0.53, respectively.

#### Questionnaire

The Japanese language version^36^ of the Balanced Inventory of Desirable Responding^37^ was used to measure the tendency to rate according to social desirability. The questionnaire consisted of 24 items, and participants were required to provide ratings using scales ranging from 1 (completely disagree) to 7 (completely agree). Tani^36^ demonstrated sufficient scale score reliability and validity and the *α* coefficient for the current study was 0.67.

#### Statistical Analysis

To examine the influence of cognitive load on intention in response to infant crying (caregiving, neglect, and physical abuse), we used paired *t* tests to compare state empathy and intention between the cognitive load conditions. We then created multilevel regression models to estimate the effects of cognitive load and state empathy on intention in response to infant crying. Data from repeated measures experiments, like the current study, are not all independent because observations are clustered within subjects; such observations within the same subject tend to be correlated and almost always have hierarchical structure^23^. When observations are correlated, it is not appropriate to conduct normal multiple regression analysis because the normal multiple regression leans heavily on the assumption of independence of observations. The violation of this assumption leads to estimate standard errors that are too small and produce Type 1 error^23^. The amount of dependence can be expressed as intraclass correlation coefficients. Lee^24^ suggested that if intraclass correlation coefficients are 0.10 or more, the effect size of intraclass correlation coefficients is large and the data has hierarchical structure. Therefore, we first obtained intraclass correlation coefficients and confirmed that the current observations had a hierarchical structure. Intraclass correlation coefficients were large (intraclass correlation coefficients ≧0.66) and we then proceeded to conduct multilevel analyses. EC and PD scores were group-mean centered, and social desirability scores, which were included in models as a control variable, were centered on their overall means. Multilevel regression models were conducted using the R 3.1.2 lmerTest package^38^,^39^. Intraclass correlation coefficients were obtained by also using the lmerTest packages. First, we conducted a null model in which no predictors were entered. Then we calculated the intraclass correlation coefficients as a ratio of subject-level error variance over the total error variance^23^. We began with a model containing only the control variable, and cognitive load and state empathy were added in Model 2; the interaction between cognitive load and state empathy was then added to examine its effect on intention. Moreover, we sought to determine whether including random intercepts for individuals and random slopes for EC and PD would improve model fit relative to that of models involving only random slopes. Likelihood ratio tests were used to evaluate model fit. We conducted simple slope analyses when interaction effects were significant. Mediation analyses were conducted to assess the mediatory effect of state empathy on the relationship between cognitive load and intention.

## Additional Information

**How to cite this article**: Hiraoka, D. and Nomura, M. The Influence of Cognitive Load on Empathy and Intention in Response to Infant Crying. *Sci. Rep*. **6**, 28247; doi: 10.1038/srep28247 (2016).

## Figures and Tables

**Table 1:** Descriptive statistics and results of ratings concerning infant crying and of a paired *t* tests comparing cognitive load conditions.

Rating	Low			High			*t*	*p*_corrected_*	Cohen’s*d*	*95% CI*
	*M*	*95% CI*	*SD*	*M*	*95% CI*	*SD*				
Providing care	15.02	14.09, 15.95	3.78	13.61	12.48, 14.73	4.59	4.45	<0.001	0.34	−0.01, 0.69
Perpetrating neglect	10.15	9.15, 11.16	4.09	11.47	10.51, 12.43	3.89	4.14	<0.001	0.34	−0.69, 0.02
Perpetrating physical abuse	6.41	5.68, 7.14	2.98	6.82	6.06, 7.58	3.09	1.83	0.358	0.14	−0.49, 0.21
EC	8.08	7.65, 8.50	1.73	7.30	6.82, 7.78	1.96	3.58	0.003	0.42	0.07, 0.78
PD	5.91	5.36, 6.45	2.20	6.26	5.71, 6.81	2.25	1.56	0.123	0.16	−0.51, 0.19

^*^p value corrected using Bonferroni correction; EC: empathic concern; PD: personal distress.

**Table 2:** Results of hierarchical multiple regression analyses.

	Model 1	Model 2	Model 3	Model 4	Model 5
**A. Intention to Provide Care**					
Fixed effects					
Intercept	7.16^***^	7.16^***^	7.16^***^	7.14^***^	7.14^***^
Social desirability	0.02	0.02	0.02	0.02	0.01
Cognitive load		−0.38^**^	−0.36^**^	−0.37^**^	−0.32^**^
EC		0.77^***^	0.6^***^	0.77^***^	0.58^***^
PD		−0.16	−0.17	−0.10	−0.10
EC × PD				−0.12	−0.29
Cognitive load × EC				−0.12	−0.1
Cognitive load × PD				0.03	−0.14
Cognitive load × EC × PD				0.36	0.69^*^
Random effects					
Intercept	3.63	3.76	3.81	3.80	3.88
EC			0.20		0.20
PD			0.17		0.22
AIC	1,010.62	928.28	932.11	933.36	919.34
Deviance	1,002.62	914.28	914.11	911.36	887.34
**B. Intention to Perpetrate Neglect**					
Fixed effects					
Intercept	5.40^***^	5.40^***^	5.41^***^	5.36^***^	5.36^***^
Social desirability	−0.01	−0.01	−0.01	−0.01	−0.01
Cognitive load		0.42^**^	0.49^**^	0.41^**^	0.45^***^
EC		−0.52^***^	−0.47^***^	−0.49^***^	−0.39^*^
PD		0.19	0.06	0.06	−0.03
EC × PD				0.23	0.12
Cognitive load × EC				−0.68*	−0.62
Cognitive load × PD				−0.05	−0.10
Cognitive load × EC × PD				−0.99^***^	−0.86^**^
Random effects					
Intercept	3.19	3.26	3.39	3.42	3.50
EC			0.76		0.66
PD			0.19		0.05
AIC	1,031.64	994.36	991.61	982.02	969.23
Deviance	1,023.64	980.36	973.61	960.02	937.23
**C. Intention to Perpetrate Physical Abuse**					
Fixed effects					
Intercept	1.15^***^	1.15^***^	0.00	1.14^***^	0.00
Social desirability	−0.01	−0.01	0.00	−0.01	0.00
Cognitive load		0.06	0.00	0.06	0.00
EC		0.03	0.00	0.03	0.00
PD		0.05	0.00	0.05	0.00
EC × PD				0.02	0.00
Cognitive load × EC				−0.12	0.00
Cognitive load × PD				−0.11	0.00
Cognitive load × EC × PD				−0.02	0.00
Random effects					
Intercept	0.03	0.03	0.03	0.03	0.18
EC			0.00		0.00
PD			0.00		0.02
AIC	950.40	954.20	963.50	960.04	969.55
Deviance	942.40	940.20	939.50	938.04	937.55

AIC: Akaike information criterion; EC: empathic concern; PD: personal distress.

**Table 3:** The items of the questionnaire concerning infant crying.

Variables	Items
Intention to provide care	“I felt like picking up the baby” “I felt like changing the diaper or bottle feeding the baby”
Intention to perpetrate physical abuse	“I wanted to slap the baby” “I wanted to shake the baby strongly”
Intention to perpetrate neglect	“I wanted to leave the baby alone” “I wanted to go to another room”
EC	“I felt concerned for the baby”
PD	“The longer I listened to the crying, the more helpless and frustrated I felt”

EC: empathic concern; PD: personal distress.
